# Prevention of inadvertent perioperative hypothermia – Guideline compliance in German hospitals

**DOI:** 10.3205/000273

**Published:** 2019-07-26

**Authors:** Philip Gabriel, Jan Höcker, Markus Steinfath, Kevin R. Kutschick, Jana Lubinska, Ernst-Peter Horn

**Affiliations:** 1 Department of Anaesthesiology, Regio Klinikum Pinneberg, Germany; 2 Department of Anaesthesiology, Klinikum Neumünster, Germany; 3 Department of Anaesthesiology and Intensive Care Medicine, University Hospital Schleswig-Holstein, Campus Kiel, Germany

**Keywords:** pre-warming, hypothermia, sublingual temperature measurement

## Abstract

Patients undergoing elective surgery are at risk for inadvertent postoperative hypothermia, defined as a core body temperature below 36°C. This study was conducted to investigate the acceptance of the recommendations of the German S3 Guideline, in particular with respect to the concept of pre-warming and sublingual temperature measurement. The main focus was to gather data concerning the postoperative core temperature and the frequency of perioperative hypothermia in patients receiving a pre-warming regime and those without. The study team investigated the local concept and measures employed to avoid inadvertent perioperative hypothermia with respect to defined outcome parameters following a specific protocol. In summary, the study hospitals vary greatly in their perioperative processes to prevent postoperative hypothermia. However, each hospital has a strategy to prevent hypothermia and was more or less successful in keeping its patients normothermic during the perioperative process. Our data could not demonstrate major differences between hospitals in the implementation strategy to prevent perioperative hypothermia in regard to the hospital size. The results of our study suggest a wide-spread acceptance, as no postoperative hypothermia was detected in a cohort of 431 patients.

## Zusammenfassung

Die 2014 publizierte AWMF S3-Leitlinie „Vermeidung von perioperativer Hypothermie“ beinhaltet verschiedene Empfehlungen und Maßnahmen, die die Inzidenz perioperativer Hypothermie (Abfall der Körperkerntemperatur unter 36°C) deutlich senken können. Ziel der vorliegenden Studie war, die tatsächliche Umsetzung dieser Empfehlungen in der klinischen Praxis zu evaluieren. Im Detail wurde untersucht, ob die Patienten präoperativ und intraoperativ gewärmt wurden und ob Temperatur-Messungen erfolgten. Der postoperative Verlauf der Körperkerntemperatur und die Häufigkeit perioperativer Hypothermien wurden bei vorgewärmten versus nicht-vorgewärmten Patienten verglichen. Zusammenfassend zeigte sich ein hohes Maß an Variationen bezüglich der durchgeführten perioperativen wärmeerhaltenden Maßnahmen. Dennoch erwies sich in den untersuchten Krankenhäusern die jeweilige Strategie zur Verhinderung der perioperativen Hypothermie als mehr oder weniger erfolgreich. Unsere Daten zeigten keine wesentlichen Unterschiede bezüglich der Umsetzungsrate bezogen auf die Krankenhausgröße. Somit legen unsere Ergebnisse nahe, dass eine weitverbreitete Akzeptanz der S3-Leitlinie vorliegt, da bei 431 untersuchten Patienten in 26 Krankenhäusern in Schleswig-Holstein keine Hypothermie auftrat.

## Introduction

Patients undergoing elective surgery are at risk for inadvertent postoperative hypothermia, defined as a core body temperature below 36°C [1], [2]. This is associated with an increased risk for wound infections, cardiac complications, and blood transfusions [3]. Patients suffering from this condition experience discomfort and shivering [4].

The German S3 Guideline for the prevention of inadvertent perioperative hypothermia was developed in accordance with the Association of the Scientific Medical Societies in Germany (AWMF) and published in 2014 [5]. It is largely based on recommendations from British, American, and Canadian guidelines [3], [6], [7]. A major difference in the German S3 Guideline is the recommendation to pre-warm surgical patients prior to administering anaesthesia [8]. This recommendation is based on recent scientific findings, which highlight the effectiveness of even brief periods of pre-warming [4], [9], [10], [11], [12].

Such recommendations, however, must be evaluated in regard to their practical implementation and acceptance, and a revision of the German S3 Guideline based on such findings is pending for 2019.

Another essential aspect of the German S3 Guideline is the recommendation to use sublingual temperature devices to assess core temperature. However, the optimal temperature measurement device is still under debate [13]. In order to be practical for regular use, the device must combine usefulness and accuracy. Furthermore, it must be possible to use it for the entire duration of the perioperative period, i.e., pre-, intra-, and postoperatively. 

This study was conducted to investigate the acceptance of the recommendations of the German S3 Guideline, in particular with respect to the concept of pre-warming and sublingual temperature measurement. The main focus was to gather data concerning the postoperative core temperature and the frequency of perioperative hypothermia in patients receiving a pre-warming regime and those without. In addition, the recommendation of sublingual temperature measurement was assessed. 

Until now, no systematically gathered data for the implementation of the guideline’s recommendations into daily practice has been available. There is some evidence that perioperative thermal management in Germany varies depending on the hospital size [14]. To this end, we analysed all German hospitals located within a circumscribed region of Northern Germany, ranging from small regional hospitals to large university clinics.

## Materials and methods

This study was approved by the Institutional Review Board of Kiel University (Chair: Prof. M. H. Mehdorn) and is registered with AZ: D 543/15. 

All hospitals in Schleswig-Holstein with 100 or more surgical procedures per year (28 of 36 hospitals) were asked to participate in the study. Therefore, the heads of the 28 anaesthesiology departments were informed about the study protocol and asked for their consent to participate. After informed consent, each hospital was visited by two members of the study team for one day within the study period of five months between January and May 2016, two years following the publication of the German S3 Guideline. The study team investigated the local concept and the measures employed to avoid inadvertent perioperative hypothermia with respect to defined outcome parameters following a specific protocol. Members of the study team were trained to properly assess sublingual temperature in postoperative patients using sublingual thermometers (Sure Temp Plus 690, Welch Allyn Inc., Skaneateles Falls, NY). 

We chose 28 visitation days within the study period. Each of these appointments was drawn by lot and randomly matched to one hospital. One day prior to investigation, each head of the anaesthesiology department was informed with the instructions to notify no one but the operating theater manager.

### Study protocol

On the morning of each investigation day, the members of the study team were introduced to the respective anaesthesiology team by the head of the department. They were shown to the recovery room of the hospital and were instructed to follow the specified routine workflow. The head of the department was interviewed in regard to the implementation of the German S3 Guideline to prevent perioperative hypothermia.

The members of the study team collected specific data from all patients following elective surgery, such as morphometrics, demographics, warming devices, temperature measurement, anaesthesia regimens, and surgical disciplines. The data were obtained from routine anaesthesiology protocols and by interviewing the involved anaesthesiologists. In addition to the routine procedures of the hospitals, the members of the study team measured sublingual temperature of all patients within the first 15 minutes following transfer to the recovery room. Collected data were entered to an Excel database. Each investigation period ended at 5 pm, without exception.

For data analysis, the hospitals were divided into three groups according to the number of hospital beds: less than 250, more than 250, or more than 450 beds.

A temperature lower than 36°C, as determined by sublingual temperature measurement, was rated as “hypothermic”, in accordance with the German S3 Guideline.

Statistical analysis was performed with the statistics software GraphPad Prism 5.0^®^ (GraphPad Software, San Diego, CA) and R 2.11.0 (R^®^ Foundation for Statistical Computing, Vienna, Austria). Continuous, normally distributed variables were analysed using one-way analysis of variance (ANOVA) and Scheffé’s F test [15].

## Results

All 28 hospitals were visited by the study team during the study period. In two hospitals, there were none or only five surgical procedures scheduled at the day of visit. As a result, these hospitals were excluded from the study. 

From the remaining 26 hospitals, data from 431 patients were collected. We had a total of 8 drop-outs due to refusal of sublingual temperature measurement or to a delay more than 15 minutes after transfer to the recovery room. Demographic and morphometric data of the patient collectives are shown in Table 1 (Tab. 1). Patients were between 3 and 96 years old, and the duration of surgery varied between 2 and 488 minutes. Within the hospital groups, no statistical differences were found.

Surgical disciplines and anaesthesiological procedures are listed in Table 2 (Tab. 2). There was a wide variation of nearly all standard surgical procedures. Minimal invasive surgery was found in 151 (35%) of the procedures. General anaesthesia was performed in 361 (84%), and regional anaesthesia in 62 (14%) of the 431 investigated patients.

The frequency of employed pre-, intra-, and postoperative forced-air warming devices and warming blankets is given in Table 3 (Tab. 3). Preoperatively, active convective warming was used in 87 of the 431 patients (20%) and in 9 of the participating 26 hospitals (35%). Preoperative warming blankets were applied in 317 (74%) of the patients in all hospitals. Intraoperative forced-air warming was used in 231 (54%) of the patients. Postoperatively, 39 (9%) of the patients were actively warmed by forced-air warming.

Temperature measurement was assessed preoperatively in 31 (7%) and intraoperatively in 179 (41%) of the patients (Table 3 (Tab. 3)). From these measurements, intraoperative hypothermia was detected in 54 (13%) of the patients.

**Figure 1:**
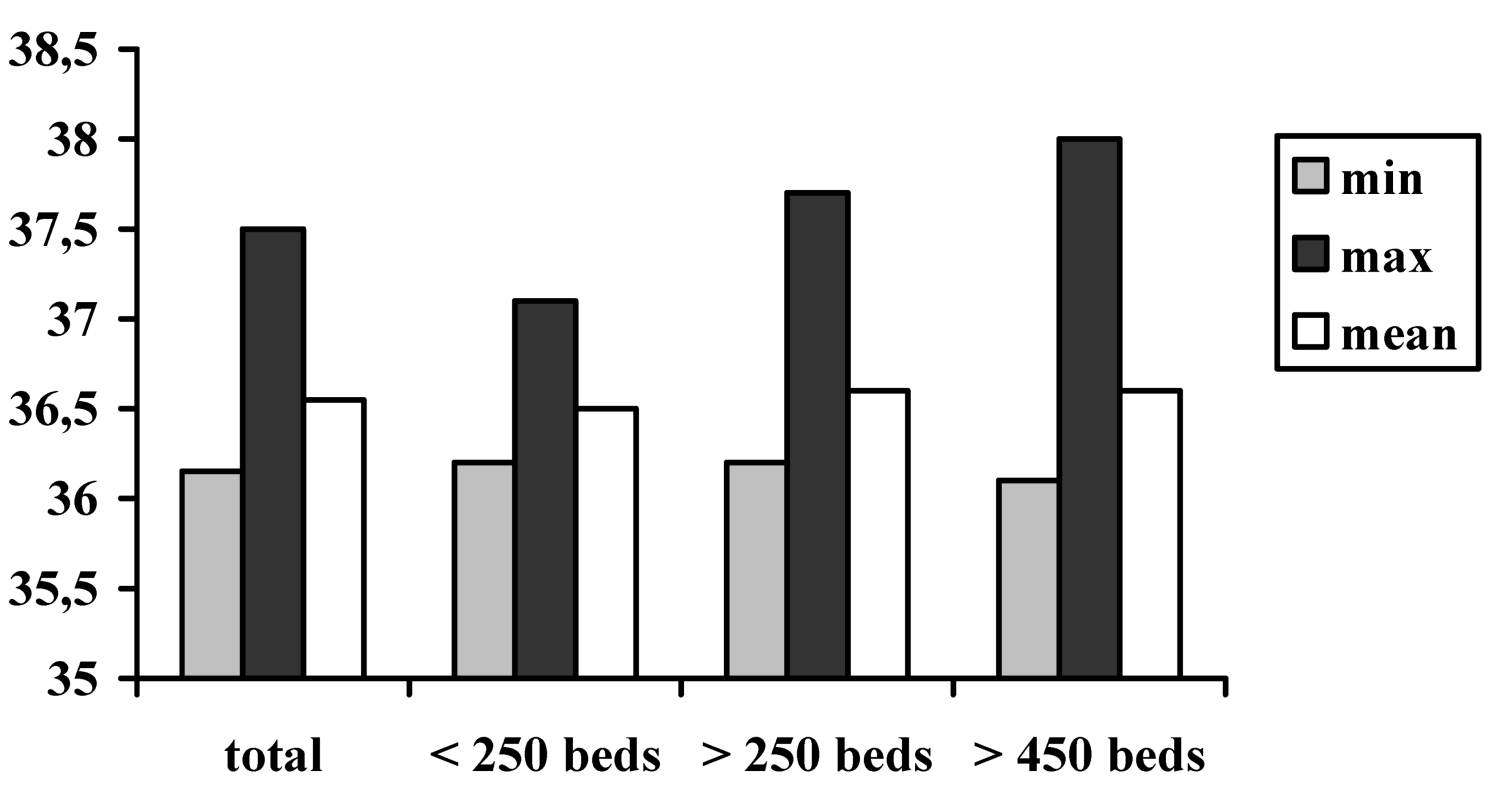
Results of the postoperative sublingual temperature (°C) measurement of the patients The lowest postoperative sublingual assessed temperature was 36°C. Therefore, none of the 431 patients were hypothermic.

The results of the measurements of sublingual temperature assessed by the study team are given in Figure 1 (Fig. 1). The mean core temperature of the patients was nearly 36.5°C upon arrival in the recovery room, and did not vary significantly between the hospital groups. The lowest sublingual temperature was measured at 36.0°C. Therefore, postoperative hypothermia was detected in none of the investigated patients.

## Discussion

We investigated the clinical application of the 2014-published German S3 Guideline to prevent perioperative hypothermia in 26 German hospitals in a circumscribed region. Our results show that none of the patients experienced postoperative hypothermia. Therefore, it can be assumed that the German S3 Guideline to prevent perioperative hypothermia has been both highly accepted and implemented in Northern German hospitals at the time of the study, two years after the Guideline’s publication.

However, preoperative active warming of the patients, one of the main recommendations in the German S3 Guideline, was used in only 20% of the patients. Our results are therefore surprising. The low rate of pre-warmed patients may be responsible for the fact that 13% of the patients suffered from intraoperative hypothermia. Furthermore, this result may have been underestimated, because temperature was measured in only 42% of the patients. Adjustment of the perioperative processes to implement pre-warming surgical patients is complicated and may be the reason for the low acceptance in the hospitals investigated. When using pre-warming, patients must arrive approximately 30 minutes earlier to the perioperative region and nurses have to start active warming devices.

In 74% of the cases, the hospitals used warming blankets to cover their patients just prior to initiation of anaesthesia. This is a well-known method to optimize patient comfort and reduce preoperative stress, although it does not appear to majorly influence the rate of perioperative hypothermia [5]. The combination of employing warming blankets, pre-warming, as well as the high frequency of intraoperative (54%) and postoperative (9%) active warming of the patients could explain the very low incidence of postoperative hypothermia we observed in our investigation.

In summary, the study hospitals vary greatly in their perioperative processes, including their implementation of different strategies to prevent postoperative hypothermia. However, each hospital has a strategy to prevent hypothermia and was more or less successful in keeping its patients normothermic during the perioperative process. Our data could not demonstrate major differences between hospitals in the implementation strategy to prevent perioperative hypothermia in regard to the hospital size. This observation contradicts a study which demonstrates that the availability of temperature-conserving devices increases with hospital size [14].

The acceptance of the German S3 Guideline recommendations to measure preoperative patient temperature and to continue temperature assessment during the surgical process was low. In only 7% of the patients, core temperature was measured preoperatively. This was a surprising result, because preoperative hypothermia of the patients should be prevented in elective surgery to avoid worse outcomes [5]. Without preoperative temperature assessment, patients are at a greater risk for hypothermia. In addition, temperature was measured intraoperatively in 42% of the patients. Today, it is inexpensive and easy to measure core temperature intraoperatively using a sublingual thermometer, a urinary bladder catheter temperature device, or other available devices. In our study, we employed a standard sublingual temperature device to assess postoperative core temperature, as recommended in the German S3 Guideline. This device demonstrates high accuracy and precision and presents results within seconds, even in patients ventilated by laryngeal masks [16]. However, sublingual temperature measurement was not used routinely in any of the investigated hospitals.

The low incidence of intraoperative temperature measurement may be the result of the high number of short surgical processes and the lack of anaesthesiologists’ knowledge of the necessity to assess core temperature during surgery. However, we found a low incidence of perioperative hypothermia even without consequent perioperative temperature assessment.

The pre-warming concept revised in the German S3 Guideline, as well as the recommendations for temperature assessment, must be critically weighed in respect to their practical acceptance and its effect on postoperative hypothermia.

According to Karalapillai et al., inadvertent hypothermia following surgical procedures is a relevant clinical problem reported in up to 46% of cases [17]. Guidelines have been published to aid in the prevention of this clinical problem. Implementation of these clinical recommendations should help in the maintenance of perioperative normothermia of the patients.

Despite the fact that the guideline recommendations of intraoperative temperature measurement and pre-warming were reported to have been only partially integrated into clinical practice in the participating Northern German clinics, no case of postoperative hypothermia was detected in our sample size of 431 patients. These positive results of partial implementation of the German S3 Guideline are very promising. Nevertheless, intraoperative hypothermia remains a problem, potentially solved by strict adherence to the German S3 Guideline.

Limitations of our study design include the reliance on a questionnaire and single-person interview with the head of the department for assessment of guideline implementation. Indeed, due to lacking implementation of guideline recommendations into daily practice, we would have expected to detect rates of hypothermia that are consistent with those in the current literature. By only informing the anaesthesiologists about the screening for the implementation of the German S3 Guideline by an external team on the day of investigation, we attempted to minimize bias. Clinical procedures could nevertheless have been influenced by knowledge of the study investigation. Furthermore, temperature measurements were taken on a single day, and the number of investigated patients was limited.

In summary, the justification of a pre-warming regimen is logical and persuasive. However, 70% of the included patients were normothermic postoperatively, without pre-warming. Therefore, at least in our study population, this strategy seems to be redundant for prevention of hypothermia. Yet in regard to the limitations of our study, further prospective trials are needed to clarify whether it is justified to resign from pre-warming as a measure of a prevention-of-hypothermia bundle.

## Notes

### Acknowledgement

We would like to thank Rosalie McDonough for her critical reading and correction of the paper.

### Competing interests

The authors declare that they have no competing interests.

## Figures and Tables

**Table 1:**
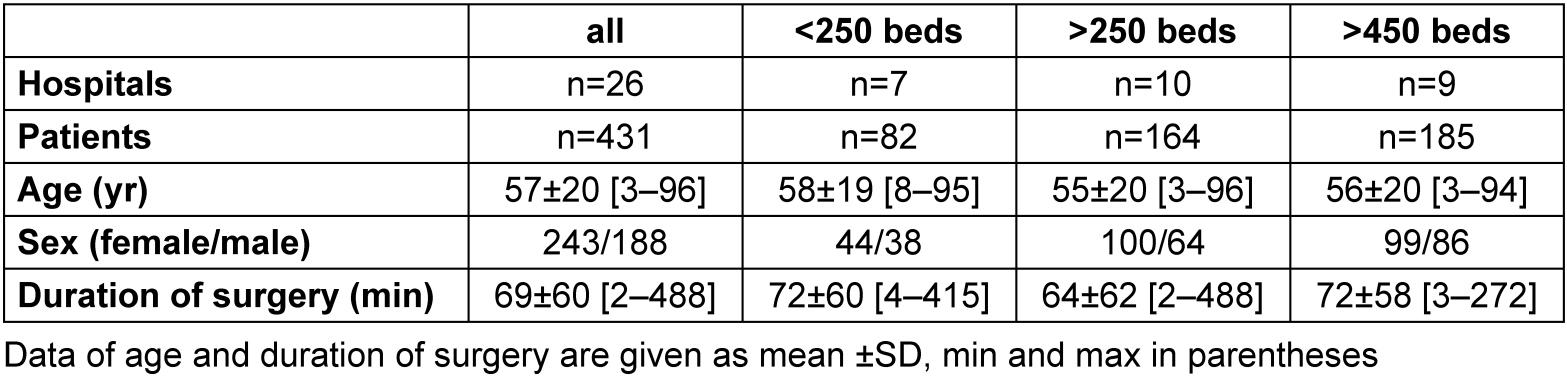
Demographic and morphometric characteristics, duration of surgery

**Table 2:**
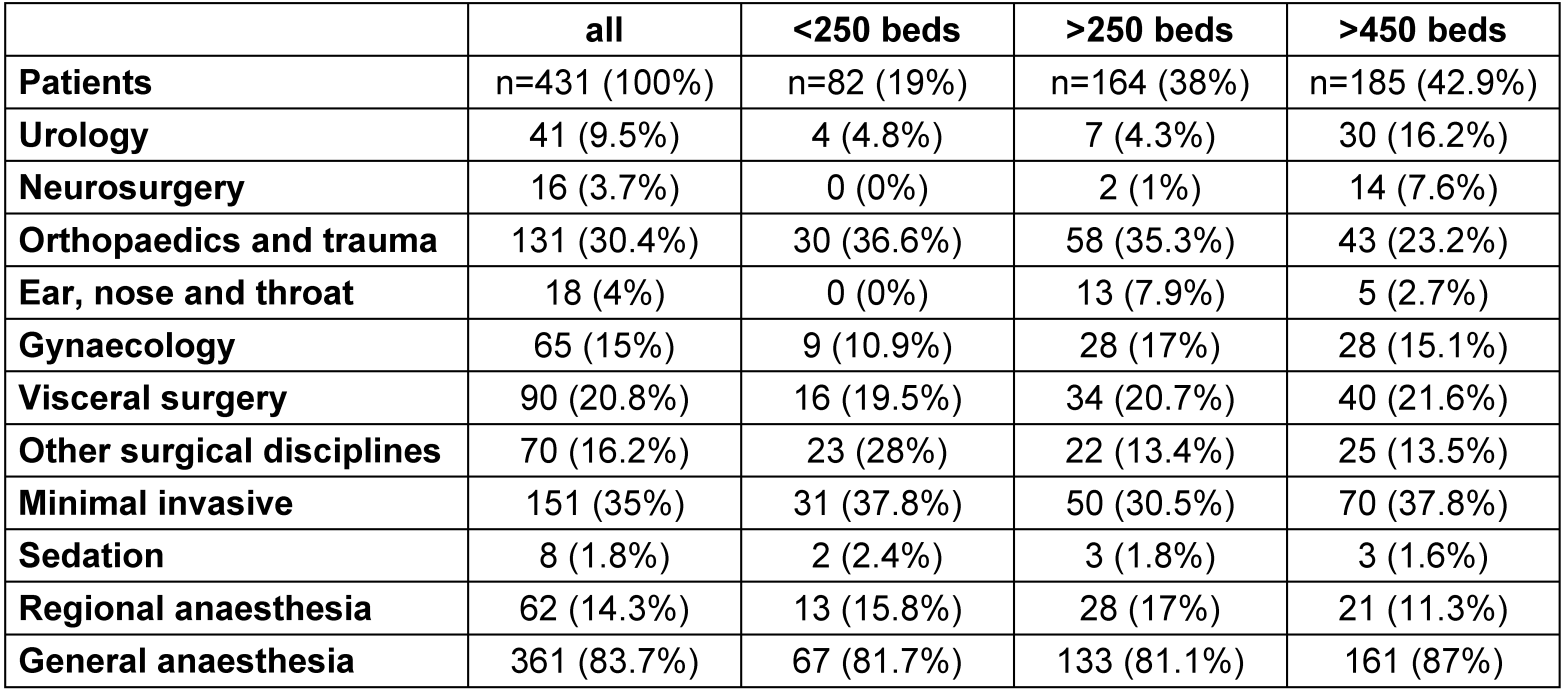
Disciplines of surgery and type of anaesthesia

**Table 3:**
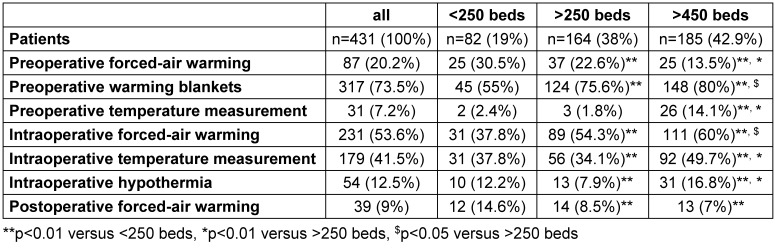
Warming devices, temperature measurement, and the incidence of intraoperative hypothermia
